# An initial study on the predictive value using multiple MRI characteristics for Ki-67 labeling index in glioma

**DOI:** 10.1186/s12967-023-03950-w

**Published:** 2023-02-11

**Authors:** Ningfang Du, Weiquan Shu, Kefeng Li, Yao Deng, Xinxin Xu, Yao Ye, Feng Tang, Renling Mao, Guangwu Lin, Shihong Li, Xuhao Fang

**Affiliations:** 1 Department of Radiology, Huadong Hospital, Fudan University, Shanghai, China; 2 Department of Neurosurgery, Huadong Hospital, Fudan University, Shanghai, China; 3 School of Medicine, University of California, San Diego, CA USA; 4 Faculty of Health Sciences and Sports, Macao Polytechnic University, Macao SAR, China; 5 Clinical Research Center for Gerontology, Huadong Hospital, Fudan University, Shanghai, China; 6 Department of Pathology, Huadong Hospital, Fudan University, Shanghai, China

**Keywords:** Glioma, Magnetic resonance imaging, Ki-67 labeling index, Diffusion-weighted magnetic resonance imaging, Apparent diffusion coefficient, Peritumoral edema

## Abstract

**Background and purpose:**

Ki-67 labeling index (LI) is an important indicator of tumor cell proliferation in glioma, which can only be obtained by postoperative biopsy at present. This study aimed to explore the correlation between Ki-67 LI and apparent diffusion coefficient (ADC) parameters and to predict the level of Ki-67 LI noninvasively before surgery by multiple MRI characteristics.

**Methods:**

Preoperative MRI data of 166 patients with pathologically confirmed glioma in our hospital from 2016 to 2020 were retrospectively analyzed. The cut-off point of Ki-67 LI for glioma grading was defined. The differences in MRI characteristics were compared between the low and high Ki-67 LI groups. The receiver operating characteristic (ROC) curve was used to estimate the accuracy of each ADC parameter in predicting the Ki-67 level, and finally a multivariate logistic regression model was constructed based on the results of ROC analysis.

**Results:**

ADC_min_, ADC_mean_, rADC_min_, rADC_mean_ and Ki-67 LI showed a negative correlation (*r* = − 0.478, *r* = − 0.369,* r* = − 0.488, *r* = − 0.388, all *P* < 0.001). The Ki-67 LI of low-grade gliomas (LGGs) was different from that of high-grade gliomas (HGGs), and the cut-off point of Ki-67 LI for distinguishing LGGs from HGGs was 9.5%, with an area under the ROC curve (AUROC) of 0.962 (95%CI 0.933–0.990). The ADC parameters in the high Ki-67 group were significantly lower than those in the low Ki-67 group (all *P* < 0.05). The peritumoral edema (PTE) of gliomas in the high Ki-67 LI group was higher than that in the low Ki-67 LI group (*P* < 0.05). The AUROC of Ki-67 LI level assessed by the multivariate logistic regression model was 0.800 (95%CI 0.721–0.879).

**Conclusions:**

There was a negative correlation between ADC parameters and Ki-67 LI, and the multivariate logistic regression model combined with peritumoral edema and ADC parameters could improve the prediction ability of Ki-67 LI.

**Supplementary Information:**

The online version contains supplementary material available at 10.1186/s12967-023-03950-w.

## Introduction

Glioma is the most common primary central nervous system (CNS) tumor in adults, which originates from neurogliocyte [1, 2], accounting for about 81 percent of malignant brain tumors [3]. Based on World Health Organization (WHO) classification, glioma has grades 1–4. The median survival of patients with glioblastoma was only 12–15 months despite aggressive, comprehensive treatment being used [4]. The fifth edition of the WHO Classification of Tumors of the Central Nervous System (WHO CNS5), published in 2021, developed a new tumor classification system and grading standards, focusing on promoting the application of molecular diagnosis in the classification of CNS tumors [5]. Accurate grade and classification of glioma are necessary for selecting the correct treatment plan since the treatment strategy for different grades of glioma is quite different [6, 7]. However, some patients with older age and multiple underlying diseases or tumors located in some critical functional areas that cannot tolerate surgery or puncture can only be treated conservatively and cannot be diagnosed pathologically. In addition, the pathological diagnosis of stereotactic biopsy or surgical resection may cause errors in pathological results due to intra-tumor heterogeneity and sampling bias [8, 9].

The proliferative activity of tumors is also an essential indicator in evaluating malignancy [10]. Differences in the proliferative activity of tumors mean that patients with the same type of tumor receiving the same treatment and dose may show different results. Ki-67 is a non-nuclear protein expressed in proliferating cells, which is related to cell proliferation, differentiation, metastasis, and apoptosis. It is mainly expressed in the S and G2 phases of the cell cycle and is a reliable, sensitive marker of tumor cell proliferation [11]. There was a significant correlation between Ki-67 labeling index (LI) and tumor grade [12]. Additionally, Ki-67 LI is not affected by factors such as age and gender. Assessment of cell proliferation activity by Ki-67 LI immunohistochemical staining can complement standard histological grading and provide meaningful therapeutic and prognostic information [13–15]. However, Ki-67 LI can only be obtained by postoperative biopsy pathology, and there is still a lack of effective preoperative predictors.

Magnetic resonance imaging (MRI) is the preferred imaging method for a variety of central nervous system tumors, including glioma [16], which can display morphological characteristics, such as peritumoral edema, location, and size of tumors noninvasively. MRI can play an essential role in the diagnosis of gliomas, especially diffusion-weighted imaging (DWI) because it has great potential to accurately distinguish glioma grades and determine the degree of malignancy, and provide a promising approach to assess the cellular structure and aggressiveness of tumors [17]. The apparent diffusion coefficient (ADC) value obtained by fitting the monoexponential model can reflect the limited degree of free water molecules in tissues [18]. Therefore, there could be potential correlations between ADC values and Ki-67 LI. Prior studies have reported that the ADC values of glioma are negatively correlated with Ki-67 LI [19, 20]. However, due to the heterogeneity of glioma, especially glioblastoma, the ADC values within glioma are uneven, so it is necessary to standardize the ADC values within the tumor to obtain more reliable results.

Surgery is the preferred initial treatment for gliomas [21]. Maximum safe tumor resection can delay disease progression, improve overall survival (OS), alleviate symptoms, and provide sufficient tumor tissue for pathological diagnosis [22]. The residual lesion caused by incomplete surgical resection is often one of the common reasons for postoperative recurrence [23]. However, the boundaries of tumor infiltration are difficult to define, and the degree of peritumoral edema (PTE) can reflect the extent of tumor cell infiltration and invasion to a certain extent. PTE usually shows a high signal on the T2 sequence but no enhancement on T1CE, which is also referred to as the non-enhancing tumor (NET) [24]. Pathological biopsy confirmed the peritumoral edema area is infiltrated by diffuse tumor cells [25, 26]. These areas are often the sites of tumor recurrence. Therefore, peritumoral edema is one of the main biological characteristics of glioma, which has an important impact on clinical prognosis and is associated with a high disability rate and high mortality rate [27]. Studies have shown that there are radiomic and pathological characteristics in the peritumoral region related to Ki-67 expression [28, 29]. These results suggest that the peritumoral edema region might be useful to be included in the construction of the Ki-67 LI prediction model for glioma.

The purpose of this study was to explore the correlations between ADC parameters and Ki-67 LI in gliomas and to predict Ki-67 LI noninvasively preoperatively combined with ADC parameters and peritumoral edema based on standard clinical MRI sequences. The developed multivariate model may provide a promising strategy for predicting glioma malignancy and proliferative activity.

## Materials and methods

### Patient selection

All patients diagnosed with glioma at our hospital between January 2016 and December 2020 were screened for the study. The inclusion criteria were as follows: (1) Meet the proven histologic diagnosis of glioma based on 2016 WHO guidelines for CNS tumors; (2) All patients underwent surgical treatment and obtained Ki-67 LI test results; (3) Preoperative MRI examination with complete data were available. The exclusion criteria were as follows: (1) Previous treatment before surgery; (2) MRI imaging quality is poor and cannot be analyzed; (3) MRI imaging or molecular results were missing, incomplete, or ambiguous; (4) Associated with other nervous system diseases, such as cerebral infarction or hemorrhage.

A total of 166 glioma patients were finally enrolled, including 92 males and 74 females, aged from 14 to 85 years old, with a median age of 53. There were 43 low-grade gliomas (12 cases in grade I and 31 cases in grade II) and 123 high-grade gliomas (18 cases in grade III and 105 cases in grade IV). All patients signed informed consent before the enhanced MRI examination according to the regulations in Huadong Hospital, Fudan University. This retrospective study was exempted from ethical review.

### MRI parameters

MR images were acquired with a 3.0-T MRI scanner (MAGNETOM Prisma; Siemens Healthineers, Erlangen, Germany). All MRI examinations included T2-weighted, T2-weighted fluid-attenuated inversion recovery (FLAIR), and T1-weighted sequences before and after administration of a gadolinium-based contrast agent and diffusion-weighted imaging. Two b values were used to calculate ADC values, b0 = 0 mm^2^/s and b1 = 1000 mm^2^/s. The patient was placed in a supine position, and a routine head scan was performed using a 64-channel combined head and neck coil. The detailed parameters of MRI scanning were provided in Additional file 1.

### Image analysis

Two radiologists with more than 10 years of experience in radiology independently conducted image analysis and processing with blind evaluation. The disagreements were resolved through consultation. The degree of peritumoral edema on MRI of the tumor was observed on the T2-FLAIR sequence, and other conventional MRI images, including enhancing images, were used as references. The degree of peritumoral edema included: no edema, mild edema (maximum diameter of edema  < maximum diameter of the tumor), and severe edema (maximum diameter of edema  ≥ maximum diameter of the tumor).

ADC parameters were measured using syngo. via workstation (Siemens Healthineers, Erlangen, Germany) by two independent observers who were blinded to pathological diagnosis, age, gender, and other information. Three different 20–30 mm^2^ regions of interest (ROIs) with the lowest visual ADC values were mapped. From these, the mean numerical ADC value of the three ROIs measurement was designated as the ADC minimum (ADC_min_). Subsequently, one ROI (ADC_mean_) was placed as large as possible to cover the largest axial tumor cross-section, excluding necrosis, cystic lesion, hemorrhage, and calcifications. Another ROI was placed in the normal white matter area in the contralateral centrum semiovale, and the measured ADC value was designated as ADC normal-appearing white matter (ADC_nawm_). Two relative ADC values are calculated according to the following formula:

1 $${\text{rADC}}_{{{\text{min}}}} \left( {{\text{relative ADC}}_{{{\text{min}}}} } \right) \, = {\text{ ADC}}_{{{\text{min}}}} /{\text{ADC}}_{{{\text{nawm}}}}$$

2 $${\text{rADC}}_{{{\text{mean}}}} \left( {{\text{relative ADC}}_{{{\text{mean}}}} } \right) \, = {\text{ ADC}}_{{{\text{mean}}}} /{\text{ADC}}_{{{\text{nawm}}}}$$

**Fig. 1:**
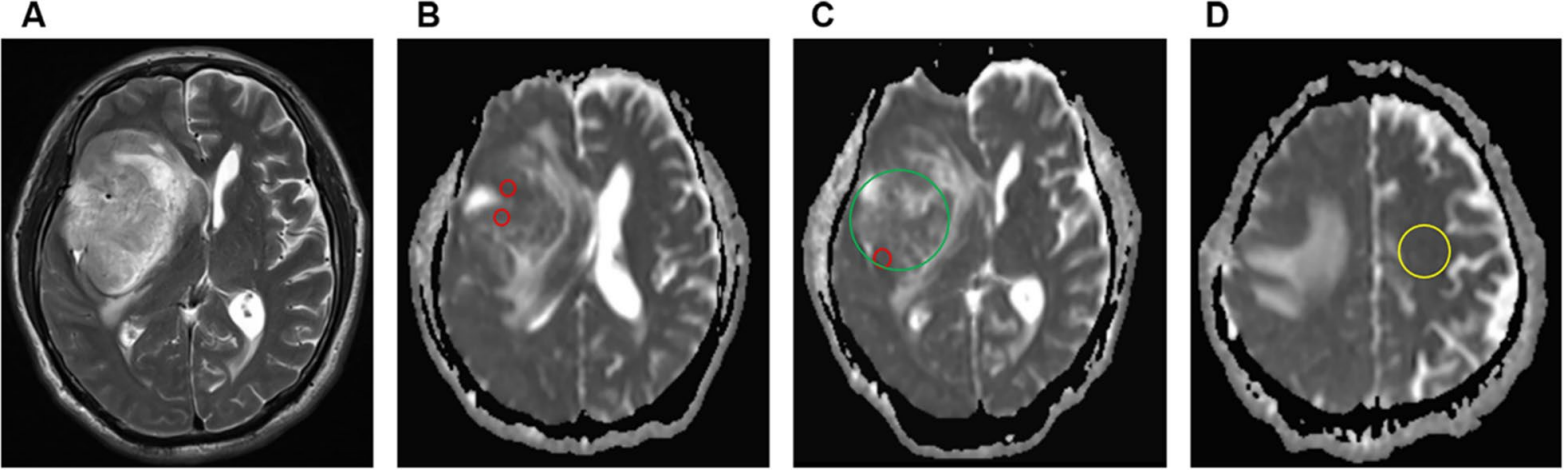
An example of apparent diffusion coefficient (ADC) measurements. **A** Axial T2-weighted image of a right frontal glioblastoma. **B**–**D** ADC maps showing the ROIs used to determine ADC_min_ (three different visually lowest ADC regions, red), ADC_mean_ (largest tumor cross-section measurement, green), and ADC_nawm_ (normal-appearing white matter in contralateral centrum semiovale, yellow)

The above ADC parameters were taken as the average values measured by 2 radiologists. The ROI placement and ADC measurement methods were referenced from a previous study by Maynard J et al. [30]. Figure 1 shows the representative images of the ROI placements.

### Histopathologic analysis

The tumors were classified as grade I–IV according to 2016 WHO guidelines for CNS tumors. The analysis was performed by two experienced neuropathologists. The samples were stained with Ki-67 immunohistochemistry and quantified according to the percentage of positive cells with the highest density in the staining area. A total of 1000 tumor cells were counted under 400 × magnification. Only immunoreactive tumor nuclei were counted, and the necrotic area and vascular endothelium were excluded. The positive percentage of tumor cells was denoted as the Ki-67 labeling index.

### Statistical analysis

SPSS 22.0 and GraphPad Prism 8 software were used for statistical analysis and mapping. Spearman correlation was used to analyze the correlations between ADC parameters and Ki-67 LI. The Shapiro–Wilk test was performed to test the normality of continuous variables. The variables in line with normal distribution were described as mean ± standard deviations (SDs) and compared by Student’s t-test. The variables not in line with normal distribution were described as median (first quartile, third quartile), and compared by Mann–Whitney U test. The categorical variables were described as number and percentage (%) and compared by Wilcoxon rank-sum test. ROC curves were used to analyze the diagnostic efficacy and cut-off points of continuous variables (ADC parameters). The sensitivity, specificity, and Youden index were calculated. Matthews correlation coefficient (MCC) was used to evaluate the binary classification performance of the unbalanced dataset. A multivariate logistic regression model was established to evaluate the level of Ki-67 LI. Variables including the degree of peritumoral edema, rADC_min,_ and rADC_mean_ were added to the multivariate logistic regression analysis. The threshold for statistical significance was set at *P* < 0.05.

## Results

### Patient demographics

The enrolled 166 patients were grouped according to their tumor grades. There was no statistical difference in gender distribution between low-grade and high-grade glioma patients (*P* = 0.172). The mean age of high-grade glioma patients (55.5 ± 14.5) was higher than that of low-grade glioma patients (38.6 ± 12.8) (*P* < 0.05). Ki-67 LI in the high-grade group [20(5,40)] was higher than that in the low-grade group [10(4,30)], and the difference was statistically significant (*P* < 0.05). The grade of glioma was positively correlated with Ki-67 LI (*r* = 0.740, *P* < 0.05). Besides, Ki-67 LI in the elderly (age  ≥ 60 years old) was higher than that of the young (age < 60 years old) (*P* < 0.05). But there was no correlation between the two (*r* = 0.279). Generally, we consider no correlation when the r value is less than 0.3.

### Correlation analysis between ADC parameters and Ki-67 LI

**Fig. 2:**
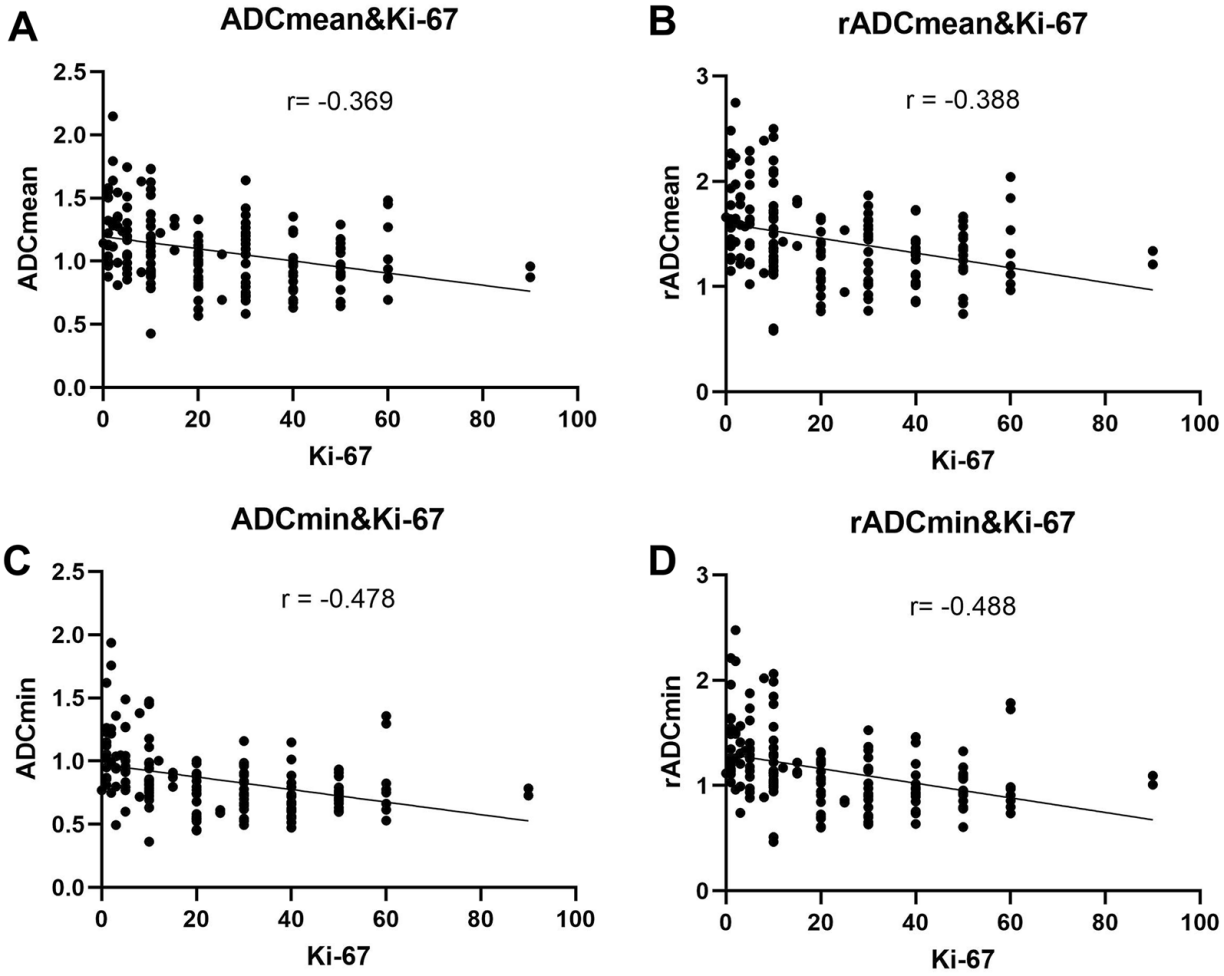
Correlations between ADC parameters and Ki-67 LI

Both ADC_min_ and rADC_min_ were negatively correlated with Ki-67 LI (*r* = − 0.478, *P* < 0.001 for ADC_min_ and *r* = − 0.488, *P* < 0.001 for rADC_min_). ADC_mean_ and rADC_mean_ were both negatively correlated with Ki-67 LI. (*r* = − 0.369, *P* < 0.001 for ADC_mean_, *r* = − 0.388, *P* < 0.001 for rADC_mean_) (Table 1, Fig. 2). ADC_min_ and rADC_min_ were more strongly correlated with Ki-67 LI than ADC_mean_ and rADC_mean_.

**Table 1:** Correlation analysis between ADC parameters and Ki-67 LI

		ADC_min_	rADC_min_	ADC_mean_	rADC_mean_
Ki-67 LI	*r *(95% CI)	− 0.478 (− 0.590–− 0.347)	− 0.488 (− 0.599–− 0.359)	− 0.369 (− 0.497–− 0.225)	− 0.388(− 0.514–− 0.246)
	*R*^*2*^	0.228	0.238	0.136	0.341
	*P*	< 0.001^***^	< 0.001^***^	< 0.001^***^	< 0.001^***^

### The cut-off point of Ki-67 LI for distinguishing LGGs and HGGs

The area under the ROC curve (AUROC) of Ki-67 LI for distinguishing LGGs from HGGs was 0.962 (95% CI 0.933–0.990). The cut-off point of Ki-67 LI was 9.5%, with a sensitivity of 92.7%, a specificity of 88.4%, and the Youden index was 0.811. So patients were divided into low (≤ 9.5%) and high (> 9.5%) Ki-67 groups.

### Comparison of ADC values and derivative parameters between low Ki-67 group and high Ki-67 group in glioma patients

The ADC_min_, ADC_mean_, rADC_min,_ and rADC_mean_ of the high Ki-67 glioma group were significantly lower than those of the low Ki-67 glioma group, and the differences were statistically significant (all *P* < 0.001) (Table 2). In all ADC parameters for differentiating the low Ki-67 gliomas from the high Ki-67 group, the diagnostic performance of standardized rADC_min_ was better than that of ADC_min_, and the standardized rADC_mean_ was higher than that of ADC_mean_. Among them, rADC_min_ had the best diagnostic performance in differentiating low Ki-67 from high Ki-67 gliomas, with an AUROC of 0.778, a sensitivity of 76.60%, a specificity of 69.75%, a MCC of 0.329, and a Youden index was 0.463 (Table 3).

**Table 2:** Comparison of ADC parameters between LGGs and HGGs

ADC parameters (× 10^−3^mm^2^/s)	Low Ki-67 group (n = 47)	High Ki-67 group (n = 119)	*t* value	*P* value
ADC_min_	1.03 ± 0.29	0.79 ± 0.21	5.89	< 0.001^***^
ADC_mean_	1.24 ± 0.28	1.03 ± 0.26	4.74	< 0.001^***^
rADC_min_	1.38 ± 0.37	1.05 ± 0.29	6.07	< 0.001^***^
rADC_mean_	1.67 ± 0.40	1.35 ± 0.35	5.03	< 0.001^***^

**Table 3:** Diagnostic performance evaluation of individual ADC parameters and the combination predictors for differentiating the low Ki-67 group from the high Ki-67 group

Parameters	AUC	95% CI	Cut-off value (× 10^−3^mm^2^/s)	Sensitivity (%)	Specificity (%)	Youden index	MCC
ADC_min_	0.770	0.693–0.848	0.86	74.47	68.91	0.434	0.306
ADC_mean_	0.714	0.631–0.797	1.11	65.96	68.07	0.340	0.211
rADC_min_	0.778	0.702–0.855	1.14	76.60	69.75	0.463	0.329
rADC_mean_	0.726	0.644–0.808	1.55	63.83	74.79	0.386	0.278
PRE	0.800	0.721–0.879	/	74.07	80.58	0.492	0.448

MCC: Matthews correlation coefficient; PRE: predictive factor

### Comparison of peritumoral edema of glioma between low Ki-67 and High Ki-67 groups

**Fig. 3:**
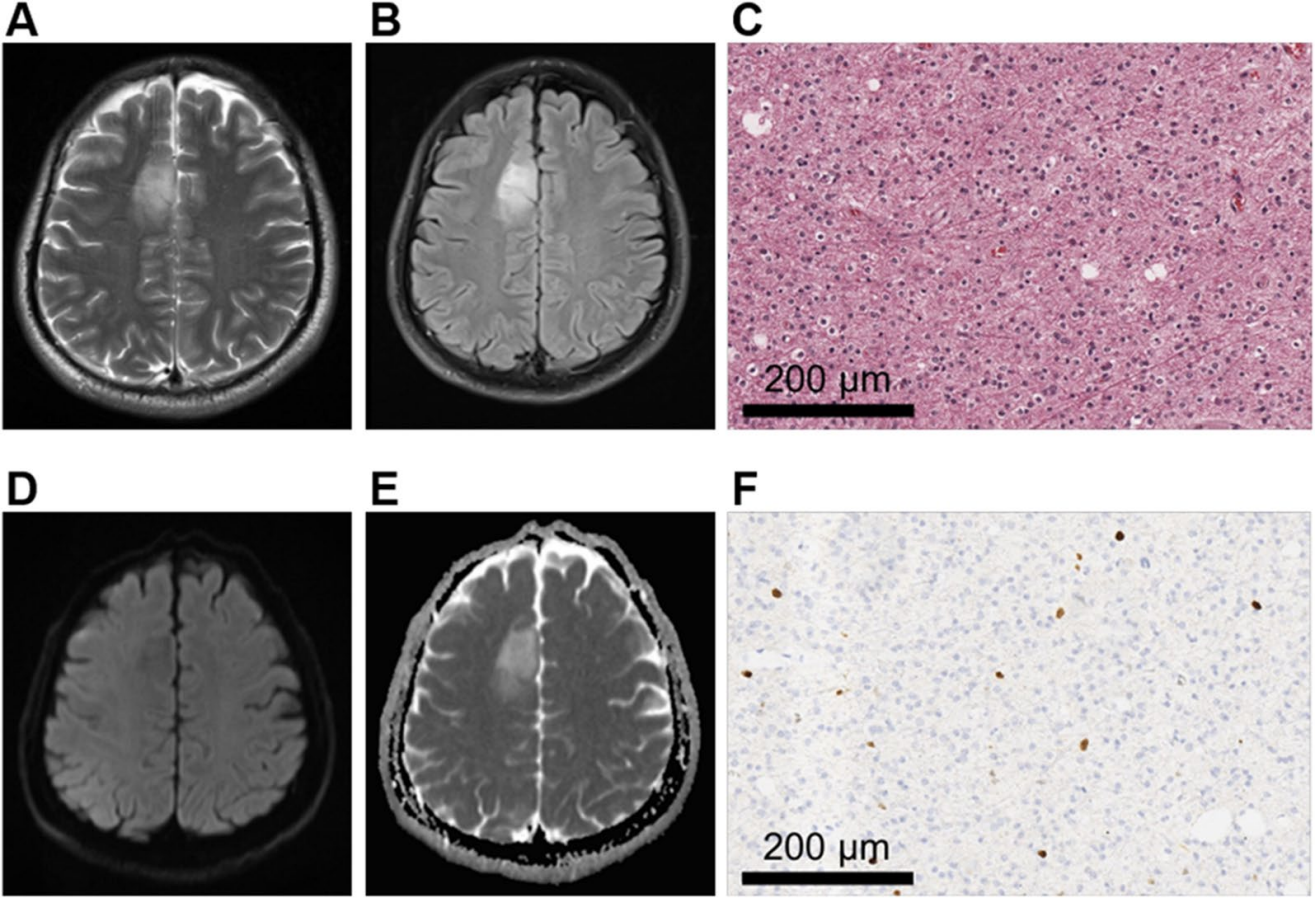
A 30-year-old male patient with right frontal oligodendroglioma, WHO grade II. **A**, **B** MRI axial T2W and T2-FLAIR sequences showed clear boundaries and no obvious edema around the tumor. **C** HE staining showed low cell density lesions and loose arrangement of cells accompanied by dense fibrous background. The cell morphology is consistent, and part of the cytoplasm is transparent. (× 200). **D**, **E** DWI and ADC images with a b value of 1000 mm^2^/s showed the tumor was unrestricted diffusion. **F** Ki-67 LI was about 4% (× 200)

**Fig. 4:**
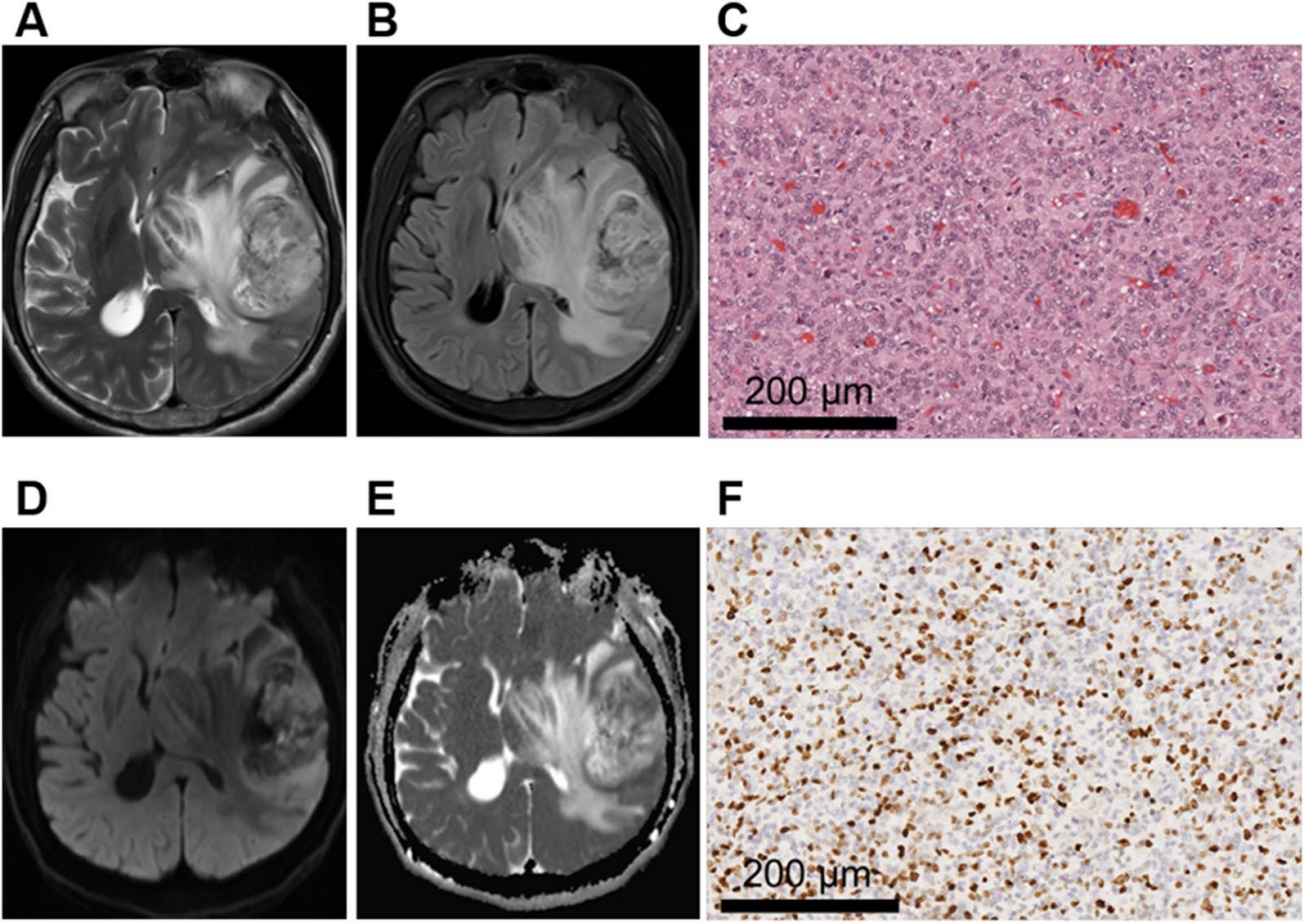
A 58-year-old male patient with left temporal glioblastoma, WHO grade IV. **A**, **B** MRI axial T2W and T2-FLAIR sequences showed tumor entity present as mixed hypersignal with severe peritumoral edema. And the tumor boundary was blurred. **C** HE staining showed significantly increased cell density, nucleoli were obvious, and karyokinesis was common (× 200). **D**, **E** DWI and ADC with a b value of 1000 mm^2^/s showed the tumor was locally obvious restricted diffusion. **F** Ki-67 was about 30% (× 200)

The degree of peritumoral edema between the high Ki-67 glioma group and the low Ki-67 glioma group was significantly different (*P* < 0.001) (Table 4). The mean rank of the low Ki-67 group was 57.83, and that of the high Ki-67 group was 93.64, suggesting that the degree of peritumoral edema was more severe in high Ki-67 LI gliomas. The representative MRI features and pathological characteristics of gliomas at different Ki-67 LI level are shown in Figs. 3 and 4.

**Table 4:** Comparison of peritumoral edema between low Ki-67 group and high Ki-67 group

Peritumoral edema	Low Ki-67 group (n = 47)	High Ki-67 group (n = 119)	*Z* value	*P* value
None	25 (53.2%)	18 (15.1%)	− 4.68	< 0.001^***^
Mild	10 (21.3%)	33 (27.7%)		
Severe	12 (25.5%)	68 (57.1%)

### Multivariate logistic regression analysis to predict Ki-67 LI

**Fig. 5:**
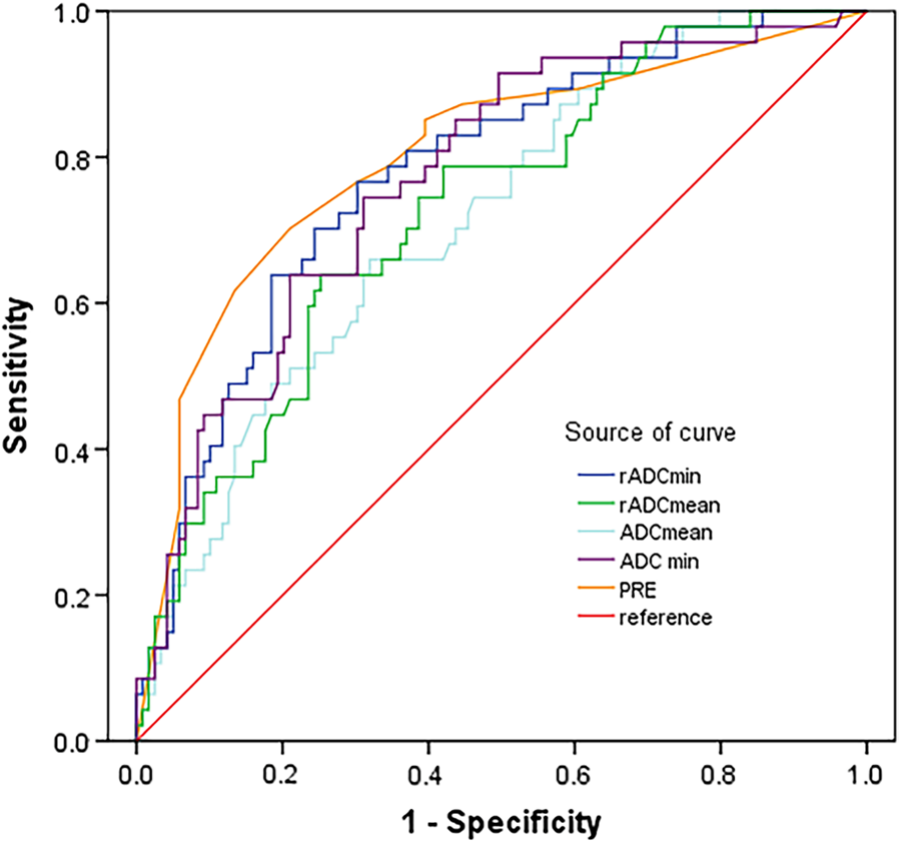
The ROC curves for predicting Ki-67 LI by the multivariate logistic regression model and ADC parameters

We next tested the multivariate model by combining all the above significant factors. Relative ADC parameters (rADC_mean_ and rADC_min_) were divided into two groups according to the optimal cut-off point in the single-factor analysis (Table 3). We found that rADC_min_ (< 1.14 × 10^–3^ mm^2^/s) and peritumoral edema were independent risk factors for predicting high Ki-67 LI (> 9.5%) (Table 5). The multivariate logistic regression model combined with ADC parameters and peritumoral edema generated a combined predictor factor (PRE). The accuracy of the multivariate logistic regression model in predicting Ki-67 LI was increased compared with that of a single ADC indicator **(**Fig. 5). The model showed an AUROC of 0.800 (95%CI: 0.721–0.879), a sensitivity of 74.07%, and a specificity of 80.58% in differentiating the low Ki-67 group from the high Ki-67 group (Table 3).

**Table 5:** Multivariate logistic regression analysis of glioma Ki-67 LI

Variables	*OR*	95%CI	*P*
rADCmin (< 1.14)	3.136	1.158–8.489	0.025^*^
rADCmean (< 1.55)	2.037	0.774–5.363	0.150
Peritumoral edema			
None	References		
Mild	4.499	1.650–12.269	0.003^**^
Evere	5.195	2.059–13.109	< 0.001^***^

^*^P < 0.05, **P < 0.01, and ***P < 0.001

## Discussion

Glioma has become the most common brain malignant tumor that affects human health and survival, bringing a heavy burden to patients and their families. Accurate assessment of tumor proliferation of glioma is essential for selecting the optimal treatment strategy. However, the current pathological method based on Ki-67 LI can only be obtained by postoperative biopsy. In this study, we developed the first non-invasive multivariate model to predict Ki-67 before surgery with over 80% accuracy. Our model includes peritumoral edema and ADC parameters from DWI that are likely to be informative of disease etiology. If replicated, this finding could have a significant impact on clinical practice by permitting patients with glioma to be stratified based on tumor malignancy before surgery and new treatment plans to be tested in clinical trials.

Ki-67 protein is a cell marker associated with ribosomal RNA transcription and cell proliferation [31]. It can be detected in the cytoplasm during interphase and migrates to the surface of chromosomes during mitosis. The Ki-67 antibody is an IgG1 monoclonal antibody, identifying a core antigen that is present in proliferating cells but absent in quiescent cells. The Ki-67 protein is expressed and detected during all phases of the cell cycle except for the early part of the G0 and G1 phases [32]. Thus Ki-67 is useful for distinguishing between growing and nonproliferating cells. Ki-67 LI was positively correlated with glioma grades and closely correlated with prognosis [33, 34]. In low-grade gliomas and ependymomas, the Ki-67 LI was inversely associated with OS [35–37]. However, the relationship between Ki-67 LI and prognosis in glioblastoma remains controversial [12, 38, 39].

DWI is a non-invasive method to detect the diffusion movement of free water molecules in tissues. ADC values can be used to indirectly understand the tightness and microstructure changes of tumor cells. Previous studies reported ADC values could effectively evaluate the grades, proliferation activity, and tumor cell density of glioma [40, 41]. In this study, ADC_min_, ADC_mean_, rADC_min_, and rADC_mean_ are all negatively related to Ki-67 LI, which is consistent with prior studies [19, 20]. In addition, among the four indicators, rADC_min_ has the highest predictive efficiency on the Ki-67 index level, with an AUROC of 0.778. This might be due to the fact that the region with the lowest ADC value in the tumor entities may best represent the part of the tumor tissue with the highest cell density and the most obvious proliferation [42]. But we also noticed that the correlation coefficients *R* were all relatively low, which may be resulting from the fact that the MRI image shows a whole glioma, and ADC measurement is also based on the whole tumor component to outline ROI. However, the Ki-67 LI is measured by pathological sections, and it only covers a small part of tumor entities, which may cause some errors, especially for tumors with high malignancy and high heterogeneity [43, 44]. Moreover, the determination of the Ki-67 index depends to some extent on the judgment of pathologists, so it cannot obtain a completely accurate and objective count. The selection and counting process of the positive cell counting area and sampling process will also affect the results. In addition, Ki-67 protein was only expressed in proliferative cells, and it would not be detected in some quiescent cells [45]. ADC measures how limited free water molecules are in tissues. Therefore, the denser the cells are, the lower the ADC value is in theory, which may also be one of the reasons for the low correlation coefficient *R* between ADC value and Ki-67 LI.

Recent studies have shown that the Ki-67 LI of high-grade gliomas is higher than that of low-grade gliomas [46, 47]. Gliomas with high Ki-67 LI are usually characterized by high cell density, nuclear atypia, pleomorphism, and heterogeneity, accompanied by vascular hyperplasia, necrosis, hemorrhage, and endothelial cell proliferation [48]. However, there is no universally accepted standard for the cut-off point of Ki-67 LI to distinguish LGGs from HGGs. In clinical practice, Ki-67 LI of 10% is usually used as a rough standard for differentiating LGGs and HGGs. According to the enrolled data, the cut-off point for Ki-67 LI to distinguish LGGs from HGGs in this study was 9.5%, which is close to the previous reports. A multicenter study in Indonesia showed significant sensitivity and specificity for distinguishing LGGs from HGGs with a Ki-67 LI cut-off point of 6.35% (*P* < 0.001) [49]. Faria MH et al. [50] found that a Ki-67 LI above 8.0% could be identified as grade IV astrocytoma, and Ki-67 LI between 1.5% and 8.0% can be identified as grade III astrocytoma (sensitivity, 0.85; specificity, 0.86), and Ki-67 LI below 1.5% can be identified as grade I and II astrocytoma. The slight difference in the cut-off point of the Ki-67 LI may be caused by the selection bias of the enrolled patients and the racial difference. In addition, inter-observer differences in the determination of the Ki-67 LI and the lack of precise guidelines can also contribute to the difference in results.

In this study, although there was no statistically significant association between Ki-67 and age, we found that Ki67 LI was higher in glioma patients over 60 years of age than in patients under 60 years of age. Older patients are often associated with a poor prognosis, previous studies have shown that IDH wild-type glioma is more common in older patients [51]. One of our previous retrospective studies also showed that age  ≥ 60 years old was an independent risk factor for predicting glioma IDH wild type and high-grade glioma [52]. Therefore, Ki-67 LI may be higher in elderly glioma patients. It may be necessary to evaluate the relationship between Ki-67 LI and glioma grades by age stratification in subsequent studies.

The peritumoral edema of glioma has always been in the spotlight. The main components of the edema tissue were scattered invasive tumor cells, reactive cells, and various blood vessel patterns [53]. The blood–brain barrier (BBB) plays a key role in the development of peritumoral edema [54]. Yong et al. found that there were indeed a large number of tumor cells in the edema area around the tumor based on proton magnetic resonance spectroscopy (MRS) [55]. The higher the Ki-67 LI, the stronger the tumor proliferation activity, and tumor oxygen consumption increases, stimulating cells to secrete vascular endothelial growth factor (VEGF), thereby promoting tumor angiogenesis. However, neovascularization lacks a basilar membrane, and vascular wall permeability increases, resulting in plasma extravasation and aggravating peritumoral edema [56]. In addition, the tumor has high proliferative activity and rapid growth rate, which can compress blood vessels in a short time and exceed normal compensatory capacity, leading to the aggravation of peritumoral edema. In this study, the degree of peritumoral edema was more severe in the group with high Ki-67 LI, which also suggested that there is a certain correlation between peritumoral edema and Ki-67 LI, both of which are related to the higher malignant degree of glioma.

The recently released 2021 guidance (WHO CNS5) has made a number of significant changes to the classification of gliomas [5]. The new guidelines require more genetic and molecular detection and have adjusted tumor taxonomy, grading, nomenclature, and many other aspects, resulting in the current failure of many pathology centers to quickly switch to the new classification. This study was a retrospective study, and all the enrolled patients were diagnosed before WHO CNS5 was published. Therefore, the WHO 2016 classification is still adopted in this study, but the new classification method of WHO CNS5 will be adopted in our future work.

In this study, we proved that all ADC parameters were negatively correlated with Ki-67 LI, obtained the Ki-67 cut-off point of 9.5% to distinguish LGGs from HGGs, and found that there were differences in the degree of peritumoral edema in gliomas with different Ki-67 LI levels. Most notably, this is the first study to our knowledge to predict Ki-67 LI by multiple MRI characteristics in glioma combining ADC parameters and peritumoral edema. The developed multivariate logistic regression model performed better than any single ADC value in evaluating Ki-67 LI.

Our study has some limitations. First, this study was a single-center retrospective study with a relatively small number of enrolled cases. Second, conventional DWI sequences were used in the study instead of multi-b values diffusion MRI imaging. The monoexponential model ignores the contribution of microvascular perfusion to ADC values at low b values. The multi-b value biexponential model IVIM (intravoxel incoherent motion) imaging can separate the perfusion fraction from the true limited diffusion state of tumor parenchyma at low b values [57]. DKI (diffusion kurtosis imaging) imaging with high b-values can reflect the complexity of the internal structure of the tumor [58]. Therefore, IVIM and DKI imaging can more accurately reflect the nature and microstructure of the tumor. But since our study is retrospective, the included patients only underwent the traditional DWI sequence that is most commonly used clinically and did not undergo advanced diffusion MRI examinations. Third, subjective judgment errors may occur since the peritumoral edema is judged based on the experience of radiologists, and there is no gold standard. Fourth, there was no complete objectified digital assessment of Ki-67 LI by immunohistochemistry. Sampling bias, the selection of positive cell counts, and the counting process itself may affect Ki-67 results.

In conclusion, we found that the ADC parameters and the degree of peritumoral edema were significantly different in gliomas with different Ki-67 levels. The multivariate logistic regression model constructed by combining ADC parameters and peritumoral edema demonstrated good predictive ability for Ki-67 LI. Once validated, this analysis may provide a simple and non-invasive imaging method for preoperative planning and prognosis assessment of patients with glioma.

## Supplementary Information

**Additional file 1: **Routine protocols of glioma.

## Data Availability

The datasets used and/or analyzed during the current study are available from the corresponding author on reasonable request.

## Abbreviations

ADC: Apparent diffusion coefficient
LI: Labeling index
ROC: Receiver operating characteristic
HGG: High-grade glioma
LGG: Low-grade glioma
AUROC: Area under the ROC curve
PTE: Peritumoral edema
MRI: Magnetic resonance imaging
CNS: Central nervous system
WHO: World Health Organization
IDH: Isocitrate dehydrogenase
DWI: Diffusion-weighted imaging
OS: Overall survival
NET: Non-enhancing tumor
FLAIR: Fluid-attenuated inversion recovery
ROI: Region of interest
SD: Standard deviations
MCC: Matthews correlation coefficient
PRE: Predictive factor
BBB: Blood–brain barrier
MRS: Magnetic resonance spectroscopy
VEGF: Vascular endothelial growth factor
